# Family carers research: What progress has been made?

**DOI:** 10.1177/02692163211037855

**Published:** 2022-02-17

**Authors:** Sheila Payne, Peter Hudson, Gunn Grande

**Affiliations:** 1 International Observatory on End of Life Care, Faculty of Health and Medicine, Lancaster University, Lancaster, Lancashire, UK; 2 Centre for Palliative Care, St Vincent’s Hospital and The University of Melbourne, Melbourne, Australia; 3 End of Life Research Department, Vrije University Brussels (VUB), Ixelles, Belgium; 4 Division of Nursing, Midwifery and Social Work, School of Health Sciences, University of Manchester, Manchester, UK

**Figure 1.**
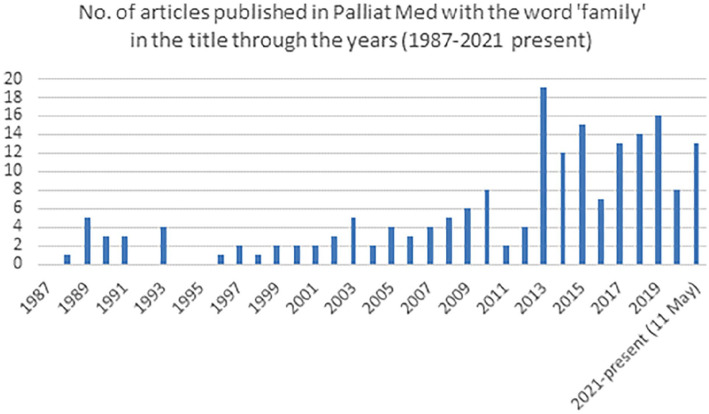
Search results of all articles in *Palliative Medicine* (since 1987) with ‘review AND family/carer/s/caregiver/s/family carer/s/family caregiver/s’ in the title.

Family carers (hereafter ‘carers’) are an essential part of palliative care provision. They often provide physical, emotional, social and financial support for the dying in the final phase of life, across all settings. Carers may also take a lead advocacy role, negotiating with multiple health and social care providers. It is therefore surprising that in years past, carers appeared to be marginalised both in the provision of support and importantly in research. Carers may have featured in research as proxy respondents for patients too ill to respond, rather than their own needs being assessed and addressed as the primary focus. Encouragingly, in more recent years, there has been a steady growth in carer research (Figure 1).

We focus this virtual special issue on reviews published in *Palliative Medicine* to provide an indication of the volume, quality and focus of research. We identified 48 reviews and selected ten based on their focus on carers in palliative care.

A range of review methods were identified including systematic^1–7^ integrative,^8^ narrative^9^ and qualitative^10^ reviews. There were three main areas of focus: (1) broad-based overviews of needs and current status of the science of carer research;^3,4,9,10^ (2) attempts to measure and define carer related concepts;^2,6,7^ and (3) attempts to design and evaluate the effectiveness of interventions to support carers to enable them to better manage their role.^1,5,8^

The focus remains predominantly on carers of people with cancer,^1,5^ we know much less about the support needs of those caring for people dying with other conditions. The literature mostly draws carers from largely homogenous populations especially those who are current users of specialist palliative care services. There is little research that focuses on carer attributes or characteristics that might impact upon their needs and ability to provide care, such as older people who may have their own health concerns, those with concurrent childcare or employment responsibilities, access to resources or health literacy. There needs to be a greater recognition of carer diversity and those from marginalised groups. The reviews demonstrate an increase in the number of assessment tools^2,7^ and interventions^1,5,8^ designed to address carers’ needs, although more intervention research applying rigorous experimental designs is advocated. More attention is needed on developing, examining and evaluating innovative interventions and the feasibility of their implementation for supporting carers and addressing their needs.

The provision of end-of-life care in the home continues to be a priority and is where the majority of family caregiving takes place.^3,4,8,9^ This may become even more important in a post-pandemic environment, where shifts in place of death may be influenced by restrictions on visiting or limited resources in institutional settings such as hospitals and hospices. The rapid increase in digital health interventions may offer new forms of support for carers or alternatively may exacerbate their feelings of isolation; we do not yet know.

Whilst the increase in research activity is encouraging; unless gaps in diversity and rigorous intervention and implementation research are addressed, there is a significant risk that needs of numerous carers will not be adequately met.
